# Healthcare Professionals’ and Policy Makers’ Views on Implementing a Clinical Practice Guideline of Hypertension Management: A Qualitative Study

**DOI:** 10.1371/journal.pone.0126191

**Published:** 2015-05-05

**Authors:** Ping Yein Lee, Su May Liew, Adina Abdullah, Nurdiana Abdullah, Chirk Jenn Ng, Nik Sherina Hanafi, Yook Chin Chia, Pauline S. M. Lai, Stalia S. L. Wong, Ee Ming Khoo

**Affiliations:** 1 Department of Family Medicine, Faculty of Medicine and Health Sciences, Universiti Putra Malaysia, Serdang, Selangor, Malaysia; 2 Department of Primary Care Medicine, University of Malaya Primary Care Research Group (UMPCRG), Faculty of Medicine, University of Malaya, Kuala Lumpur, Malaysia; 3 Klinik Perubatan Keluarga SL Wong, Klang, Selangor, Malaysia; University of Glasgow, UNITED KINGDOM

## Abstract

**Introduction:**

Most studies have reported barriers to guideline usage mainly from doctors’ perspective; few have reported the perspective of other stakeholders. This study aimed to determine the views and barriers to adherence of a national clinical practice guideline (CPG) on management of hypertension from the perspectives of policymakers, doctors and allied healthcare professionals.

**Methods:**

This study used a qualitative approach with purposive sampling. Seven in depth interviews and six focus group discussions were conducted with 35 healthcare professionals (policy makers, doctors, pharmacists and nurses) at a teaching hospital in Kuala Lumpur, Malaysia, between February and June 2013. All interviews were audio-recorded, transcribed verbatim and checked. Thematic approach was used to analyse the data.

**Results:**

Two main themes and three sub-themes emerged from this study. The main themes were (1) variation in the use of CPG and (2) barriers to adherence to CPG. The three sub-themes for barriers were issues inherent to the CPG, systems and policy that is not supportive of CPG use, and attitudes and behaviour of stakeholders. The main users of the CPG were the primary care doctors. Pharmacists only partially use the guidelines, while nurses and policy makers were not using the CPG at all. Participants had suggested few strategies to improve usage and adherence to CPG. First, update the CPG regularly and keep its content simple with specific sections for allied health workers. Second, use technology to facilitate CPG accessibility and provide protected time for implementation of CPG recommendations. Third, incorporate local CPG in professional training, link CPG adherence to key performance indicators and provide incentives for its use.

**Conclusions:**

Barriers to the use of CPG hypertension management span across all stakeholders. The development and implementation of CPG focused mainly on doctors with lack of involvement of other healthcare stakeholders. Guidelines should be made simple, current, reliable, accessible, inclusive of all stakeholders and with good policy support.

## Introduction

Clinical practice guidelines (CPGs) are statements that include recommendations intended to optimise patient care that are informed by a systematic review of evidence and an assessment of the benefits and harms of alternative care options [1]. Many countries are producing their own CPGs tailoring to their own local setting and resources [2–4]. The Guidelines International Network (GIN) has a collection of 6642 documents (including 2809 separate guidelines) from 96 organisations and 75 countries in its library [5]. CPGs can be used to measure and improve the quality of clinical practice [6].

In a span of 2 decades, the number of CPGs has risen a hundred-fold from 70 in 1990 [7] to 7508 in 2012 [8]. For hypertension alone, there are 114 listed guidelines in PubMed [9]. Despite this overwhelming number of CPGs, implementation of guidelines has proved to be challenging. A systematic review and other studies on guideline utilisation have shown “leakage” at all steps, from awareness to adherence, resulting in non-adherence [10, 11]. Barriers to guideline adherence include physician factors such as lack of awareness, non-familiarity, disagreement, lack of self-efficacy and low outcome expectancy as well as the inertia of previous practice and external barriers [12].

Cabana et al. also noted that barriers differed from setting to setting. The effectiveness of an intervention to improve adherence to guidelines is therefore not generalisable and must first consider the barriers that are present [12]. The Canadian Hypertension Education Program (CHEP) is a knowledge translation programme that provides updated standardised recommendations and clinical practice guidelines in hypertension management. This programme targets various healthcare professionals in clinical and community settings [4]. It emphasises the importance of multiple partnerships, stakeholders, supporters and multidisciplinary participants to ensure dissemination of key messages to a broader audience. CHEP is stated to be the likely contributor to the improvement of hypertension management and reduction of related disease in Canada [13]. The success of this programme’s implementation was related to the identification of barriers and addressing related issues in the local setting [14]. In the UK, a round table discussion between representatives from primary and secondary care settings on the 2011 NICE/BHS hypertension guidelines identified various challenges in guideline implementation. These include the accuracy of data entry and blood pressure measurement, patients’ concordance with antihypertensive drug regimens and resistant hypertension. Various initiatives were planned to support practices in meeting the challenges of managing primary hypertension. These include more stringent audit standards of data entry, the development of a patient decision aid relating to the guideline and an ambulatory blood pressure monitoring (ABPM) hypertension register [15]. In Netherlands, the recommendation of a report on guidelines development also noted that a preliminary analysis of the target group, and setting was necessary before implementation activities [10, 12].

Systematic reviews have reported barriers to guideline usage mainly from the doctors’ perspective; few studies have reported the perspectives of other stakeholders [11, 12, 16]. Involvement of policymakers and other members of the healthcare team may improve the implementation of the CPG in actual practice. This study aimed to determine the views and barriers to adherence of a national clinical practice guideline on management of hypertension from the perspectives of policymakers, doctors and allied healthcare professionals.

## Methods

### Study design and setting

The study design was qualitative using in-depth interviews and focus group discussions to explore views, perceptions, beliefs and values of healthcare providers (HCPs) on the use of CPGs [17, 18]. This study is part of a larger research project, which aims to develop and evaluate an intervention to improve adherence to the Malaysian CPG on management of hypertension. This study was conducted at a university teaching hospital in Kuala Lumpur, Malaysia. This teaching hospital has about 1000 beds and an attached primary care clinic that serves as a gatekeeper for other disciplines and admissions.

### Clinical practice guideline

We used the Malaysian CPG on management of hypertension (3rd edition 2008), as this version was in use at the time of this study [19]. This guideline was developed for all healthcare providers involved in the management of hypertension in adults, elderly, pregnant women and children. Its aim was to guide healthcare providers on the diagnosis, assessment, investigations and management of patients with hypertension in adults, pregnant women, children and patients with specific co-morbidities such as chronic kidney diseases, metabolic syndrome, diabetes and stroke. The stakeholders involved in the development of the CPG were nephrologists, cardiologists, family physicians, obstetrician/gynaecologists, endocrinologists, general physician/clinical pharmacologists, paediatricians, epidemiologists and pharmaco-economists.

### Participants, recruitment and sampling

We recruited 41 participants using purposive sampling [20]. Seven in-depth interviews were conducted with policymakers, and six focus group discussions (4–6 participants each group) with 28 doctors (4 primary care physicians, 4 medical officers, 11 primary care trainees) and allied health professionals (4 pharmacists and 5 primary care nurses) between February and June 2013. The policymakers recruited were specialist doctors, pharmacists and nurses who had an additional administrative role in forming hospital policies or were members of the drug and therapeutic subcommittee (which is responsible for the hospital formulary and medication expenditure). The primary care nurses were involved in the clinical management of patients with hypertension such as the measurement of blood pressure and weight. Participants were approached by an email or telephone call. After agreeing to participate, an in-depth interview or focus group discussion (FGD) was arranged. Composition of the FGD was grouped according to the participants’ profession to facilitate interaction. Policymakers were individually interviewed to overcome scheduling difficulties. Two policymakers who refused to participate because of busy schedule and time constraints were replaced by their colleagues [21]. Table 1 outlines the demographic data of the participants.

**Table 1:** Participants demographic characteristic.

Category	Characteristic	Number (%)
**Professions**	Policy maker	7 (20.0)
	Pharmacist	4 (11.4)
	Nurse	5 (14.3)
	Family Medicine Trainee	11 (31.4)
	Medical Officer	4 (11.4)
	Family Medicine Specialist	4 (11.4)
**Age range (years)**	24–35	16 (45.7)
	36–45	8 (22.9)
	46–55	5 (14.3)
	56–65	6 (17.1)
**Gender**	Male	13 (37.1)
	Female	22 (62.9)
**Ethnicity**	Malay	18 (51.4)
	Chinese	8 (22.9)
	Indian	7 (20.0)
	Others	2 (5.7)
**Years in Practice (years)**	≤5	5 (14.3)
	6–10	14 (40.0)
	11–15	2 (5.7)
	16–20	3 (8.6)
	21–25	1 (2.9)
	26–30	2 (5.7)
	31–35	7 (20.0)
	36–40	1 (2.9)

### Data collection

A topic guide was used to facilitate the interviews (S1 Appendix). The topic guide was developed based on literature review and the health belief model [22, 23]. It was modified slightly to cater for different stakeholders (primary care doctors, pharmacists and nurses, and policymakers). Open-ended questions were used to interview the participants and prompts were only used when important issues did not emerge spontaneously during the interview. Examples of questions asked were “What are your views on the (Malaysian) hypertension CPG? And “What are the barriers or reasons for not using CPG?”. These open-ended questions allow participants to give their views on the main aim of this study to explore their views and barriers of CPG use and adherence. The duration of each session ranged from 60 to 90 minutes. Written informed consent for the interviews and audio recording was obtained from the participants. Five experienced academic primary care researchers [EMK (female,), SML (female), PYL (female), CJN (male) and SSLW (female)] conducted the interviews. Field notes on non-verbal cues and interview dynamics were taken by an assistant. All interviews were audio-recorded, transcribed verbatim and checked. Data from in-depth interviews, focus group discussions and field notes were used for triangulation. Non-verbal cues were recorded in field notes to verify and validate participants’ responses. Interviews and analyses were performed in an iterative manner until no new themes emerged. Recruitment was stopped when researchers agreed that the analysis had reached thematic saturation. This was achieved after seven individual interviews and six focus group discussions.

### Data analysis and validation

Data were analysed using a thematic analysis approach. Themes were derived inductively from the data. Three researchers coded two transcripts (one focus group discussion and one in-depth interview) independently and a list of free nodes (themes) was created.

Subsequently, the themes were merged to form categories. This framework was then used to code (label) another two transcripts. The coding was then compared for inter-rater consistency and discrepancies. Any disagreements were resolved through consensus. The final framework was used to code the rest of the transcripts. Any new themes emerged were added to the list with the consensus of the research team. NVivo 10 software was used to manage the data.

Researchers involved in the analysis were primary care physicians and a pharmacist [PYL (MMed), SML(PhD), EMK(MD), NSH (PhD), AA (MMed), NA (MMed), PLSM (PhD) and CJN (PhD)]. All researchers were conscious of their personal views and biases about hypertension CPG adherence. The team had constant reflection and open discussion throughout the analysis. The quotes that best captured the essence of the themes were extracted for presentation in the results. All participants were invited through emails to comment on the summary of the results and to check the validity of our data interpretation [24]. Analyses were further refined from the feedback obtained from participants.

### Ethics approval

Ethics approval was granted by the University of Malaya Medical Centre Medical Ethics Committee. Ref No. 890.14

## Results

Two main themes and three sub-themes emerged from this study. The main themes were (1) variation in the use of CPG and (2) barriers to adherence to CPG. The three sub-themes for barriers were issues inherent to the CPG, systems and policy that is not supportive of CPG use, and attitudes and behaviour of stakeholders.

### Stakeholders’ awareness and use of CPG

Participants ranged from guideline users to non-users. The main users of the CPG were primary care doctors. They became aware of the CPG during their postgraduate training in primary care medicine. They used it because it was perceived as relevant to their practice.

*“Yeah*, *I think we sort of know that (about CPG) during medical student times*. *Yeah but we didn’t know how important it was*, *and we didn’t know how to update ourselves until I joined the master programme and then the senior said*, *“Oh*, *you can download (the CPGs) from this site*.*”*

34-year-old family medicine trainee with 8 years of practice

Pharmacists were mainly partial users of guidelines. They were more familiar with international guidelines such as the seventh report of the Joint National Committee on Prevention, Detection, Evaluation, and Treatment of High Blood Pressure, American hypertension guideline (JNC 7) and the National Institute for Health and Care Excellence (2011) Hypertension: clinical management of primary hypertension in adults, British hypertension guideline (NICE) guidelines as these were referred to during their training. Their use of the local CPG was limited to medication budget planning.

Non-users of the CPG were mainly the nurses and some policy makers. There were nurses who were unaware of the existence of the CPG. Most nurses viewed CPG to be irrelevant to their practice.

*“In my opinion*, *(the CPG) may be not suitable for nurses to use because a lot of information are more for doctors*. *It is too detail*. *Especially*, *this is about medications and type of medications*. *Like beta blockers and all others*, *of course we have learned that during our student time*, *but for work*, *we do not specify to which group of drug… For nurses*, *I think it (the CPG) has to be simpler*.*”*

37-year-old nurse with 14 years of practice

Policymakers who were specialist doctors such as cardiologist or nephrologist mainly used international guidelines.

*“(I used) NICE (National Institute for Health and Care Excellence (2011)*. *Hypertension*: *clinical management of primary hypertension in adults) guidelines of 2012—obviously you’re going to use the latest guidelines available*. *Most of the guidelines in Malaysia were (not) written that way*. *It wasn’t like (US or European) guidelines whereby they have quite detailed kind of discussion about the studies involved*.*”*

33-year-old policy maker/nephrologist with 8 years of practice

Other policymakers did not use the CPG but it was important for healthcare providers to standardise care. Similar to the practising pharmacists, policymakers who were pharmacists mainly used the CPG for medication budget planning.

*“But it just*, *it’s a guide*. *I think (for) policymakers*, *those who are going to decide how much medication to buy*, *I think that is something that we can actually use (the guideline)*.*”*

52-year-old policy maker/pharmacist with more than 20 years of practice

### Barriers

Several barriers to CPG use emerged. These were categorised into three sub-themes: (1) issues inherent to the CPG, (2) systems and policy barriers and (3) attitudes and behaviour of stakeholders.

#### Issues inherent to the CPG

Participants felt that there were several barriers related to the CPG itself. The CPG was perceived to be irrelevant to those who were not doctors, the format was not user friendly and it was not seen to be as reliable or as current as international guidelines.

*“I think after we have read through so many CPG*, *I find that one very frustrating part for doctors is because they are so small (the font)*, *they write in long lengthy words*. *My ideal CPG is (not) just it can provide a guide but preferably they do it in a very simplified manner*, *let’s say screening*, *you just put it in one page*, *nice table*, *straight to the point only*, *and is easy for us to remember*, *is easy for us to read through*, *and is easy for us implement”*

34-year-old family medicine trainee with 9 years of practice

*“Yes*, *I’m aware*, *I think the CPG in Malaysia still largely depends on the*, *EAC (European Association of cardiology) guideline or the NICE (National Institute for Health and Care Excellence*, *2011)*, *Hypertension*: *clinical management of primary hypertension in adults guideline*. *Or even the JNC 7 (The seventh report of the Joint National Committee on Prevention*, *Detection*, *Evaluation*, *and Treatment of High Blood Pressure)*. *We basically we are trying to adopt other countries’ (guidelines)*, *we do (have) some our own local data*. *But*, *I must say 80–90% is a direct photocopy (from international guidelines)*.*”*

43-year-old policy maker/cardiologist with 18 years of practice

Strategies suggested to improve adherence to guideline included the provision of sections relevant to other stakeholders such as pharmacists, nurses and patients. These sections include topics such as use of home monitoring by patients, medication counselling by pharmacists, patient education and the roles of different stakeholders. The format can be improved by reducing the use of text and increasing the use of diagrams and tables. It was also suggested that the guidelines should be updated yearly and used in the training of healthcare professionals.

#### System and policy barriers

Barriers identified within the system and policies were restricted availability of anti-hypertensive drugs, lack of infrastructure for medication storage and work environment. Prescribers felt restraint to prescribe anti-hypertensive drugs because of the drug restriction policies. However, pharmacist and policymakers viewed drug restriction policies to be important in controlling medication cost. One issue that was frequently raised was the policy on the need to prescribe angiotensin-converting enzyme inhibitors before angiotensin receptor blockers. This policy was perceived to be overly rigid by the doctors.

*“But sometime we don’t (think the system support flexibility)*, *even though we want to start ARB (angiotensin receptor blockers) because patients have taken ACE (angiotensin-converting enzyme) inhibitors from private general practitioners with problem of side effects*, *but because this (pharmacy) guideline*, *we (have to) start with ACE inhibitor first (in this institution)*.*”*

63-year-old medical officer with 36 years of practice

Another issue was the non-availability of combination drugs for patients with compliance issues due to the lack of medication storage space and cost constraints.

*“…on one hand we know the advantage of having a combo (combination drug) because it will improve compliance*. *But on the other hand we have problem to probably keep so many types of drug*. *…Storage*, *yes*, *is also one of problem*.*”*

52-year-old policy maker/pharmacist with more than 20 years of practice

Work environment influenced the use of the guidelines. A policy maker viewed that doctors, being in a teaching hospital, wanted more professional freedom to prescribe novel or new drugs not used routinely in practice (currently, doctors are restraint by hospital policies in the categories of drugs they can prescribe due to budget constraint).

*“So*, *we restrict the prescriber on prescribing it*. *So*, *in essence you actually forcing the prescriber to comply into the guideline*, *let’s see if there is anything more appropriate as a first line rather than jumping straight away to ARB (angiotensin receptor blockers) …Not ideal*, *but it help you in terms of cost-effectiveness*.*”*

29-year-old pharmacist with 4 years of practice

The CPG was not available or accessible to the nurses. Doctors felt that more time was needed to implement aspects of CPG such as giving advice on lifestyle changes, which was difficult in a busy clinic. Strategies that were suggested by participants to overcome systems and policy barriers included utilising technology to facilitate CPG accessibility and providing protected time for practice.

*“So when they’re looking (for the CPG) then it should be readily*, *easily*, *accessible to them*. *I think we should do the e-library or something like that*.*”*

32-year-old pharmacist with 7 years of practice

*“(Interviewer-Does the workload actually affect whether you can practice according to the guideline*?*) Definitely*, *definitely*. *Because if you give (me) another 3 minutes…*.*I don’t think so lah*. *I can’t do anything much except for (asking) is he taking the medication and continue medications*, *if the BP is within the range*, *I am not interested to do anything else because err hundreds waiting outside*. *But if I have more time*, *then definitely I can go venturing into his diet and style of living*.*”*

40-year-old family medicine trainee with 15 years of practice

#### Attitudes and behaviour of stakeholders

Adherence to CPG appeared to be dependent on the attitude of the users. Doctors tended to follow the practice of senior medical specialists, which at times differed from the guideline. These discrepancies resulted in non-adherence to the CPG.

*“Last time (when) I was in internal med then*, *yeah*, *we follow the CPG but it also depends on what your*, *your specialist think*, *and different discipline*, *they prefer different medications*.*”*

34-year-old family medicine trainee with 9 years of practice

Strategies suggested to improve these barriers were to incorporate training in the use of the local CPG in undergraduate and postgraduate curriculum, the provision of incentives such as rewarding continuous professional development (CPD) points on CPG adherence and linking CPG adherence to key performance indicators.

*“The awareness should be from undergraduate days*. *So that they understand that we have to manage patient who have hypertension properly and these are the guidelines for the teaching…yeah*.*”*

42-year-old family medicine specialist with 18 years of practice

*“Basically for myself*, *because the emphasis to use this guideline is not there*, *as I said there’s no CPD (continuous professional development) point to actually… contribute to whole thing (to use the CPG)*.*”*

28-year-old pharmacist with 4 years of practice

Doctors in general perceived that patients were part of the barriers to CPG adherence. Patients were perceived to resist the initiation and optimisation of anti-hypertensive drugs. Doctors had difficulty in adhering to the CPG recommendation in patients with multiple co-morbidities and patients’ beliefs and misconceptions. Participants suggested that patients should be educated on blood pressure and its management.

*“Yes… one of the common one is*, *they all (patients) think that anti-hypertensive can cause kidney failure which are not true*, *second one the younger generations*, *not younger*, *I mean the younger hypertensive males*, *they think that anti-hypertensive can cause erectile dysfunction*. *Not completely wrong because beta blockers we do know that they can cause erectile dysfunction*, *but is they don’t know that this not the first line that we use nowadays*, *and then some of them*, *they would say that “oh*, *I’ve to take life long*, *so I don’t want to start it*.*”*

34-year-old family medicine trainee with 9 years of practice

## Discussion

When this research was first conceived, the aim was to obtain the views from all stakeholders involved in the management of hypertension in order to develop an effective intervention to improve CPG adherence. However, we found that not all stakeholders used the local CPG. Primary care doctors used (including family medicine trainees, primary care medical officers and family medicine specialist) CPG as it was viewed to be relevant. In non-users, the fundamental obstacles to the use of CPG were lack of awareness and perceived non-relevance of the guidelines. Other studies have also shown that lack of awareness of the CPG is a major barrier to CPG use [5, 6]. Any strategy to improve adherence would have to address these issues. The CPG was perceived to be not relevant because it did not appear to cater for the actual day-to-day practice of nurses who do not prescribe, policymakers who do not manage patients, and specialists who mainly manage patients according to complications and their respective specialty. The partial users including the pharmacists and hospital specialists have little trust on the reliability of the local guidelines when compared to international guidelines such as those from NICE and JNC. The local CPG was perceived to be not as current as international guidelines [2, 3, 19]. The participants perceived that the CPG was targeted only for primary care doctors although the stated aim in the CPG was “to provide a clear and concise approach to all health care providers on the current concepts in the management of hypertension” [19]. The findings of this study indicate the importance of revising the guidelines to cater to the needs and practice of all HCPs including nurses, doctors, pharmacists and policymakers [25, 26].

The format of the CPG was perceived to hinder its acceptance and usability. The participants in this study suggested that a simpler format with summary of main recommendations would facilitate the use of CPG. A qualitative study with Spanish physicians had also found that professionals preferred brief and simple formats [27]. An abridged version of the local CPG used in this study is available [28]. It is uncertain whether criticisms of the format could be due to the lack of awareness of the abridged version of the CPG by the participants or if an even simpler version is necessary.

Participants recommended the use of separate sections to cater for the different groups of HCPs to be incorporated into the guideline. In the NICE hypertension guideline, there is a section on patient’s perspective and a summary for patients and caregivers [2]. However, there were also no separate sections dedicated for nurses and pharmacists in other guidelines [2, 29]. The addition of specific sections on the role of the different stakeholders including patients, nurses and pharmacists would make the CPG relevant to all. The local CPG development committee was said to reflect all the different stakeholders, but notable exclusions were nurses and patient representatives. In contrast, these stakeholder groups were represented in the development committees of JNC 7 and NICE [2, 3]. This could be addressed in future guideline updates.

Lack of policies supporting the use of CPG was a major barrier to the implementation of guidelines. This affected the HCPs’ awareness, adherence and attitudes to the use of guidelines. There is also a restraint of professional freedom in drug prescription due to local prescribing policy. In addition, CPG was perceived to be inaccessible to certain group of healthcare professionals, especially the nurses. There is also time constraint in implementing the CPG, especially the lifestyle therapeutic intervention as this is viewed to be a challenge in a busy primary care clinic with heavy patient load and short consultation time. Most participants felt that an institutional policy on the CPG was warranted and important, yet there has been no movement or plan to structure a policy. They suggested to implement a policy for all health care providers—necessitating practices for implementation such as dissemination and accessibility of the guidelines, standardisation of drugs use according to the guidelines, training to improve awareness, knowledge and skills, audit of clinical practices and quality improvement programmes. This is consistent with recommendations from other studies [25–27]. In a study by Green LA et al., guidelines implementation strategies focused on barrier reduction such as reducing physicians’ time pressure and task complexity were the only ones that improved performance. Education and incentives were not found to have discernible effect [30]. A system that can better utilise other primary care team members such as dieticians and nurse practitioners to assist in patients’ lifestyle modification is desirable. However, we propose that any corrective intervention should first rectify the issues inherent within the guidelines that may lead to scepticism by the healthcare providers (such as outdated and unreliable information) before changes in policies are implemented. Otherwise, the attitude of mistrust by the healthcare providers would hinder the use of the CPG [30–32].

### Strengths and limitations

This study has included the views from all groups of healthcare providers in the management of hypertension. The qualitative study design enabled an in-depth investigation into the factors that hindered the adherence of a guideline. However, the design of this study will not uncover the factors, especially those which the participants are not aware of. One example is therapeutic inertia; this would be better detected by an audit of prescribing practices. Another limitation is that patients, an important stakeholder group, were not included as participants in this study.

### Implication to practice

National guidelines should involve all stakeholders and be relevant, evidence based and up-to-date [33]. National and institutional policies are required to support the implementation of the guidelines. Further studies should also look into patient views about adherence to clinical guidelines.

## Conclusions

Barriers to the use of CPG on management of hypertension span across all stakeholders. Despite the established importance of multidisciplinary approach to health care, especially in the management of chronic diseases such as hypertension, the implementation of CPG has focused mainly on doctors. There is a lack of involvement of other healthcare stakeholders from development to implementation. The stakeholders in this study wanted to be involved in the development and implementation of CPG. We believe that a more multidisciplinary approach is necessary for successful implementation of a CPG. Guidelines should be made simple, current, reliable, accessible, inclusive of all stakeholders, and with a good policy support. Such comprehensive strategies may improve adherence to guidelines.

## Supporting Information

S1 Appendix Topic guide of interview for primary care doctors. (DOCX)

S2 Appendix COREQ checklist. (DOC)
