# Supplementary material for: Healthcare Professionals’ and Policy Makers’ Views on Implementing a Clinical Practice Guideline of Hypertension Management: A Qualitative Study
Source: PLoS One. 2015 May 5;10(5):e0126191. doi: 10.1371/journal.pone.0126191 (PMC4420249; doi:10.1371/journal.pone.0126191)
Supplement: S1 Appendix — (DOCX) [file pone.0126191.s001.docx]

**Appendix 1**

**Topic guide for primary care doctors**

**Objective:**

- To explore the views on **needs, barriers and facilitators** faced by **primary care doctors** when implementing the CPG on hypertension in a primary care clinic

The focus group discussion will be for doctors who are involved in managing patients with hypertension in the primary care clinic and it will be:

1. Primary care clinic doctor – doctors (comprising medical officers, family medicine academic specialists and trainees)

**Participant Information sheet (PIS) – age, gender, designation**

Preamble:

- Ice breaking (refreshment)
- Explain the objectives
- Go through the PIS and explain what is an FGD
- Explain no right or wrong answers
- It is the opinions that we are interested in
- Feel free to agree or disagree with each other
- Explain the need for audio-recording
- Obtain consent for the FGD and audio-recording

This FGD is about the **needs, barriers and facilitators** faced by doctors with regards to the implementation of the CPG on hypertension in a primary care clinic

**Views**

1. What do you think of the care for hypertension in your clinic? May need more triggers as this is a very general question eg do they (doctors) need nurses and other allied health professionals to help measure BP, weight, go through diet eg salt reduction ,exercise . Why can’t docs do it themselves as there are not enough such allied health care professionals esp in small clinics …rural areas etc
2. What do you think of the control of hypertension among patients in your clinic?
3. What do you think of CPGs? Let’s focus on hypertension CPG
4. What are the hypertension guidelines you are aware of? Which do you use, why? If not, why not?
5. Have you heard of the Malaysian CPG? if not, why not? Are you aware where you can get it from? Have you read it fully? If not why not?
6. What are your views on the (Malaysian) hypertension CPG?

- Do you think the CPG is useful? If Yes, which part in particular do you find useful? If no,why not?

**Aim of CPG**

What do you think the aim of CPG on hypertension should be?

**Practice/needs**

1. Do you have a copy of the CPG on hypertension in your clinic?
2. If not, do you think there should be a CPG on hypertension in your clinic?
3. Do you carry out your practice according to the CPG?

- if yes, do you follow the guidelines strictly or partially? If partial, which part do they follow?
- if not, why not? If partially why?

**Content of CPG**

What do you think of the content of the CPG? Which version of CPG are you referring to? [1^st^ edition -1998, 2^nd^ edition -2002, 3^rd^ edition -2008)

- if the content is irrelevant, where do you think the sources of content for the CPG should be from?
- Are you aware of the sections of the CPG? (history, examination, investigation, diagnosis, treatment )
- how useful is each of the section? If not useful, why not?
- Which section do you find most useful? Why?
- Which section do you find least useful or need improvement? How can these sections be improved

**Development**

- Who do you think should be involved in the development of CPG guideline?
- Who should be the target group of the CPG?
- How to improve the accessibility of the CPG?

**Barriers**

What are the barriers or reasons for not using CPG? (no prompt first)

- Content?
- Pharma influence?
- system factor– workload, time, resources, accessibility of CPG, cost and availability of drugs
- doctor factor? personal feeling, think that it is not applicable to the patients that he or she is seeing? Perception that patient will not want to increase their medications?
- Patient factor? adherence and compliance to medication, blood taking, medication, time constraint, share decision making
- Clinic setting? lack of support from the staff

In your opinion, what would be the most effective way to improve the use of the guidelines?

Do you think the Malaysian CPG can be applied or used in your clinic?

Other guidelines

- Do you read other guidelines? How do you choose to read a certain guideline?
- Does having to read so many guidelines impede you to read each carefully and thoroughly?

**Facilitators**

If you use CPG, who introduced the use of CPG in your clinic?

Where do you get CPG from?

When do you implement CPG in your practice?

How can adherence to CPG be improved?

**How can we improve the implementation of CPG?**

Content?summarized the important point?

Clinic setting?

The policy maker?

The doctor?

The allied healthcare staff?

Patient factor?

Mass media?

Campaign?

Teaching session?Seminar, targeted group?(e.g. housemen?)

**Strategies**

1. Do you think the doctors are competent to practice the guidelines? if not, why not? What do they need to make them competent?

What are the other strategies that can be done to improve the implementation of CPG?

- Do you think these strategies are feasible?
- How likely is it going to be done? Why?

**Others?**

Suggestion overall structure:

1. Opening questions on CPGs as a whole then focusing on hypertension CPG
2. Practice – whether they use the CPG; if so, how they do they use it?
3. Ask for comments on the CPG in more details (assuming most read the Malaysian CPG)
4. Facilitators
5. Barriers
6. Needs
